# Automatic Classification of Early Parkinson's Disease with Multi-Modal MR Imaging

**DOI:** 10.1371/journal.pone.0047714

**Published:** 2012-11-09

**Authors:** Dan Long, Jinwei Wang, Min Xuan, Quanquan Gu, Xiaojun Xu, Dexing Kong, Minming Zhang

**Affiliations:** 1 Department of Radiology, the Second Affiliated Hospital, Zhejiang University School of Medicine, Hangzhou, China; 2 Center of Mathematical Sciences, Zhejiang University, Hangzhou, China; 3 Department of Mathematics, Zhejiang University, Hangzhou, China; Wake Forest School of Medicine, United States of America

## Abstract

**Background:**

In recent years, neuroimaging has been increasingly used as an objective method for the diagnosis of Parkinson's disease (PD). Most previous studies were based on invasive imaging modalities or on a single modality which was not an ideal diagnostic tool. In this study, we developed a non-invasive technology intended for use in the diagnosis of early PD by integrating the advantages of various modals.

**Materials and Methods:**

Nineteen early PD patients and twenty-seven normal volunteers participated in this study. For each subject, we collected resting-state functional magnetic resonance imaging (rsfMRI) and structural images. For the rsfMRI images, we extracted the characteristics at three different levels: ALFF (amplitude of low-frequency fluctuations), ReHo (regional homogeneity) and RFCS (regional functional connectivity strength). For the structural images, we extracted the volume characteristics from the gray matter (GM), the white matter (WM) and the cerebrospinal fluid (CSF). A two-sample t-test was used for the feature selection, and then the remaining features were fused for classification. Finally a classifier for early PD patients and normal control subjects was identified from support vector machine training. The performance of the classifier was evaluated using the leave-one-out cross-validation method.

**Results:**

Using the proposed methods to classify the data set, good results (accuracy  = 86.96%, sensitivity  = 78.95%, specificity  = 92.59%) were obtained.

**Conclusions:**

This method demonstrates a promising diagnosis performance by the integration of information from a variety of imaging modalities, and it shows potential for improving the clinical diagnosis and treatment of PD.

## Introduction

Parkinson's disease (PD) is the most common movement disorder and the second most common neurodegenerative disease [1]. For early PD patients, the most obvious symptoms mainly include resting tremor, bradykinesia and rigidity [2]. In many patients, subsequent cognitive and behavioral problems may arise, with dementia commonly occurring in the advanced stages of the disease [3]. Current diagnostic criteria for PD rely on the presence of motor signs. The patient is always clearly diagnosed with PD at the advanced stage. Moreover, any neuroprotective therapy initiated at such a late stage may have fewer substantial effects on the disease progression. Thus, it is crucial to find out some valid and objective biomarkers to distinguish early PD patients from the healthy population.

Over the past two decades, various objective measures have been adopted for the differential diagnosis of PD, including a range of olfactory, electrophysiological and neuropsychological tests [4]. However, the most developed area in providing an objective assessment is neuroimaging [5]. Many imaging methods have been employed for the diagnosis of PD, the most common being positron emission tomography( PET) and single-photon emission computed tomography (SPECT) [5]. These two imaging techniques use a variety of radioactive tracers to quantitatively assess the areas of brain blood flow, glucose metabolism and brain pharmacology to identify markers of PD. Several studies have demonstrated that these two methods are powerful tools for the diagnosis of PD [6], [7]; however, due to their invasiveness and high cost, there is a need for more inexpensive alternative techniques for early diagnosis of PD.

Recently, resting-state functional magnetic resonance imaging (rsfMRI), a non-invasive technology with high spatial and temporal resolution, has been used to study abnormal brain function in a variety of neuropsychiatric diseases. Compared with the healthy controls, regional homogeneity (ReHo) in the putamen and cerebellum differed significantly and it was correlated with the Unified Parkinson's Disease Rating Scale (UPDRS) in PD [8]. In addition, another technique (voxel-based morphometry) has enabled the analysis of structural brain changes from the perspective of the whole brain. Moreover, some studies have found decreased GM volume in the frontal, temporal and parietal brain areas, hippocampus and anterior cingulate cortex [9], [10], [11]. Regression analyses revealed a negative linear relationship between the total UPDRS-III scores and the regional GM volume in the left supplementary motor area, left primary motor cortex, right ventral premotor cortex and bilateral dorsal premotor cortex in idiopathic PD patients [12]. Taken together, these studies have shown that the structural and functional changes observed in PD patients significantly correlate with the severity of the disease. Presumably, we would be able to distinguish early PD patients from a healthy population if we could efficiently integrate the structural and functional information.

**Figure 1:**
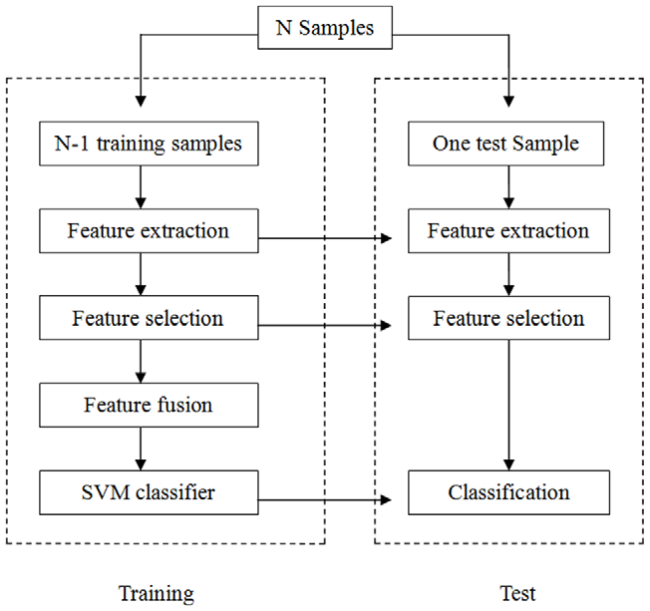
A flowchart of the multi-model method for classification.

To test our hypothesis, we first extracted multi-level characteristics (ALFF, ReHo and RFCS) from the fMRI data and extracted the GM, WM and CSF volume from the structural data. A feature selection algorithm was performed to select the most discriminative features, which were then fused together. Finally, based on the fused features, we used a machine learning technique to construct a classifier for the early diagnosis of PD (Fig. 1).

## Materials and Methods

### Subjects

Nineteen right-handed patients and twenty-seven normal volunteers participated in this study after signing an informed consent form. The age and gender differences between the two groups were tested using a two-sample t-test and a χ^2^ test, respectively, and no significant differences were observed between the groups (p>0.05). The patients were recruited from the Department of Neurology, and the normal volunteers were recruited from Zhejiang University and the communities. The study was approved by the Medical Ethics Committee of the Second Affiliated Hospital, Zhejiang University School of Medicine.

The diagnosis of PD was based on the medical history, neurological examination, response to dopaminergic drugs, and the exclusion of other neuropsychiatric diseases. Anti-parkinsonian medicine was terminated at least 12 hours prior to the imaging scans. The scores obtained from the Unified Parkinson's Disease Rating Scale (UPDRS) and the Hoehn and Yahr Scale (H&Y) were assessed for all subjects prior to scanning. All subjects were diagnosed at an early stage (H&Y I-II). Table 1 lists the clinical data for the PD group.

**Table 1:** Clinical details of all patients.

No.	Gender	Age	Disease duration	UPDRS	H&Y
1	F	60	24	11	1.5
2	M	57	24	36	2
3	F	68	9	17	2
4	F	48	12	22	1
5	F	72	18	23	1
6	F	50	6	18	1
7	F	54	36	33	1.5
8	M	52	36	26	2
9	F	47	12	32	2
10	M	38	12	27	1.5
11	M	54	12	26	1
12	M	65	6	27	2
13	F	44	12	9	1
14	F	71	4	23	1
15	F	62	6	11	1
16	M	58	10	39	2
17	M	59	12	33	2
18	F	47	24	50	2
19	M	57	24	12	1
Mean(SD)		55.9(9.2)	15.7(9.7)	25(10.7)	

Abbreviations: UPDRS, Unified Parkinson's disease Rating Scale; H&Y, Hoehn and Yahr Scale; M, male; F, female.

**Table 2:** Regions of interest (ROIs) included in the AAL-atlas.

Regions	Abbreviations	Regions	Abbreviations
Superior frontal gyrus, dorsolateral	SFGdor	Middle frontal gyrus, orbital part	ORBmid
Middle frontal gyrus	MFG	Inferior frontal gyrus, orbital part	ORBinf
Inferior frontal gyrus, opercular part	IFGoperc	Superior frontal gyrus, medial orbital	ORBsupmed
Inferior frontal gyrus, triangular part	IFGtriang	Gyrus rectus	REC
Rolandic operculum	ROL	Insula	INS
Supplementary motor area	SMA	Anterior cingulate and paracingulate gyri	ACG
Superior frontal gyrus, medial	SFGmed	Median cingulate and paracingulate gyri	DCG
Cuneus	CUN	Posterior cingulate gyrus	PCG
Lingual gyrus	LING	Parahippocampal gyrus	PHG
Superior occipital gyrus	SOG	Temporal pole: superior temporal gyrus	TPOsup
Middle occipital gyrus	MOG	Temporal pole: middle temporal gyrus	TPOmid
Inferior occipital gyrus	IOG	Olfactory cortex	OLF
Fusiform gyrus	FFG	Hippocampus	HIP
Superior parietal gyrus	SPG	Amygdala	AMYG
Inferior parietal, but supramarginal and angular gyri	IPL	Caudate nucleus	CAU
Supramarginal gyrus	SMG	Lenticular nucleus, putamen	PUT
Angular gyrus	ANG	Lenticular nucleus, pallidum	PAL
Precuneus	PCUN	Thalamus	THA
Paracentral lobule	PCL	Precentral gyrus	PreCG
Superior temporal gyrus	STG	Calcarine fissure and surrounding cortex	CAL
Middle temporal gyrus	MTG	Postcentral gyrus	PoCG
Inferior temporal gyrus	ITG	Heschl gyrus	HES
Superior frontal gyrus, orbital part	ORBsup	Vermis_10	Ver10
Cerebelum_Crus1	CERcr1	Cerebelum_Crus2	CERcr2
Cerebelum_3	CER3	Cerebelum_4_5	CER45
Cerebelum_6	CER6	Cerebelum_7b	CER7
Cerebelum_8	CER8	Cerebelum_9	CER9
Cerebelum_10	CER10	Vermis_1_2	Ver1_2
Vermis_3	Ver3	Vermis_4_5	Ver4_5
Vermis_6	Ver6	Vermis_7	Ver7
Vermis_8	Ver8	Vermis_9	Ver9

### Data acquisition

All data were acquired using a 3.0 T GE Signa MR scanner equipped with a birdcage coil. Foam padding and earplugs were used to limit head movement and reduce scanner noise for the subject. During the data acquisition, the subjects were instructed to keep their eyes closed, but not to fall asleep, and to relax their minds and move as little as possible. The functional images were collected axially using an echo-planar imaging (EPI) sequence. The imaging parameters were as follows: repetition time = 2000 ms; echo time = 30 ms; slices = 25; thickness = 5 mm; gap = 1 mm; field of view = 240×240 mm^2^; resolution = 64×64; and flip angle = 80°. The scan lasted for 390 s. Three-dimensional axial Fast Spoiled Gradient Recalled (3D-FSGPR) images were collected using the following parameters: TR/TE = 5100 ms/1.2 ms; FOV = 24×24 cm; matrix = 256×256; slices = 124; thickness = 1.2 mm; and space  = 0 mm.

**Table 3:** Classification performance of the single metrics and multi-modal combinations.

Metrics	Accuracy	Sensitivity	Specificity
All modal combination	86.96%	78.95%	92.59%
ReHo+ALFF+RFCS	73.91%	57.89%	85.19%
ALFF+RFCS	67.39%	47.37%	81.48%
GM+WM+CSF	80.43%	84.21%	77.78%

**Table 4:** The number of features retained in the multi-model method per fold.

Fold	ReHo	ALFF	RFCS	GM	WM	CSF
1	1	5	6	4	21	22
2	2	2	7	4	12	23
3	2	2	5	5	18	22
4	6	6	8	5	14	23
5	2	2	6	4	9	18
6	1	2	7	4	13	19
7	2	2	4	3	12	22
8	2	1	5	3	12	21
9	1	1	6	5	17	22
10	4	4	4	7	8	20
11	1	3	3	4	10	23
12	1	2	6	3	10	19
13	2	5	5	4	15	23
14	1	6	6	4	21	19
15	2	3	2	5	20	26
16	2	2	7	3	8	22
17	2	1	3	4	9	20
18	2	1	4	5	8	23
19	6	4	5	5	4	20
20	3	4	5	4	13	22
21	2	2	8	4	12	19
22	2	4	8	3	11	20
23	1	1	3	4	14	20
24	2	1	6	5	10	19
25	1	1	3	3	13	19
26	2	2	8	3	9	21
27	2	3	5	6	9	23
28	2	3	6	4	9	21
29	2	3	4	5	14	21
30	3	4	5	5	15	24
31	4	4	4	5	19	25
32	1	2	8	5	12	23
33	2	1	4	4	10	24
34	3	5	5	4	14	19
35	2	2	6	4	13	20
36	1	1	3	5	17	22
37	3	2	7	4	15	21
38	3	3	6	6	12	23
39	3	4	5	5	16	21
40	2	5	4	5	16	26
41	1	3	5	5	12	21
42	2	3	5	6	14	25
43	2	5	6	5	11	19
44	1	2	6	4	10	20
45	2	5	5	6	9	21
46	2	1	3	4	12	19

### Data preprocessing

All functional imaging data preprocessing was performed using the Statistical Parametric Mapping (SPM8, http://www.fil.ion.ucl.ac.uk/spm) and Data Processing Assistant for Resting-State fMRI (DPARSF) programs [13]. The ALFF and ReHo were calculated using the Resting-State fMRI Data Analysis Toolkit (REST, http://rest.restfmri.net) and the RFCS calculation program developed by our group (MATLAB R2009a).

**Figure 2:**
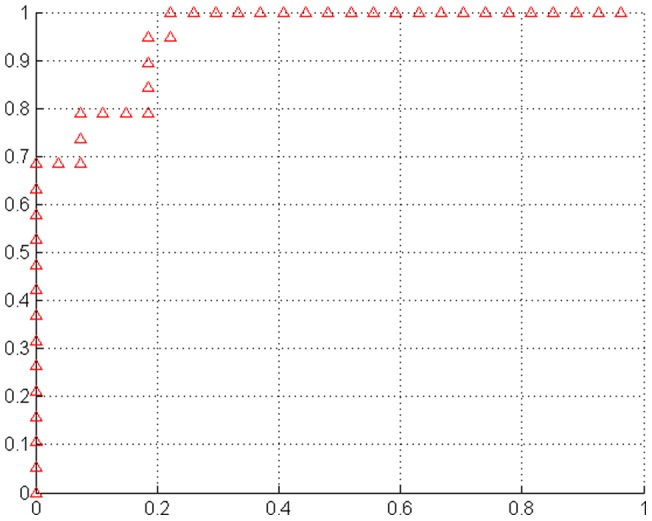
Classification performance of the multi-model method. The ROC curve of the classifier. The area under the ROC curve was 0.951.

**Figure 3:**
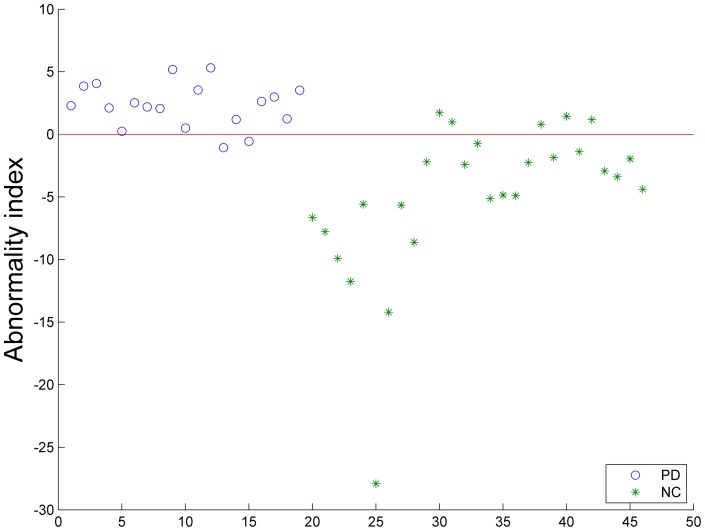
Scatter plot of the abnormality index-scores of all subjects. Positive scores represent subjects classified in the PD group and negative scores represent subjects classified in the NC group.

Preprocessing of the rsfMRI data was performed as follows. We discarded the first 10 volumes, taking into account factors that affect the instability of the initial MR signals and the adaptation of the subjects to the circumstances. The remaining images were then corrected for the within-scan acquisition time differences between slices and further realigned to the first volume to correct for interscan head motions. Individuals with an estimated maximum displacement in any direction greater than 2 mm or a head rotation greater than 1° were discarded to minimize movement artifacts in this study. The motion-corrected functional volumes were then spatially normalized to the MNI template and re-sampled into 2-mm isotropic voxels [14]. Next, temporal band-pass filtering (0.01 Hz–0.1 Hz) was performed on the time series for each voxel to reduce the effect of the low-frequency drifts and high-frequency physiological noise [15]. We then calculated the ALFF, ReHo and RFCS as described below.

**Figure 4:**
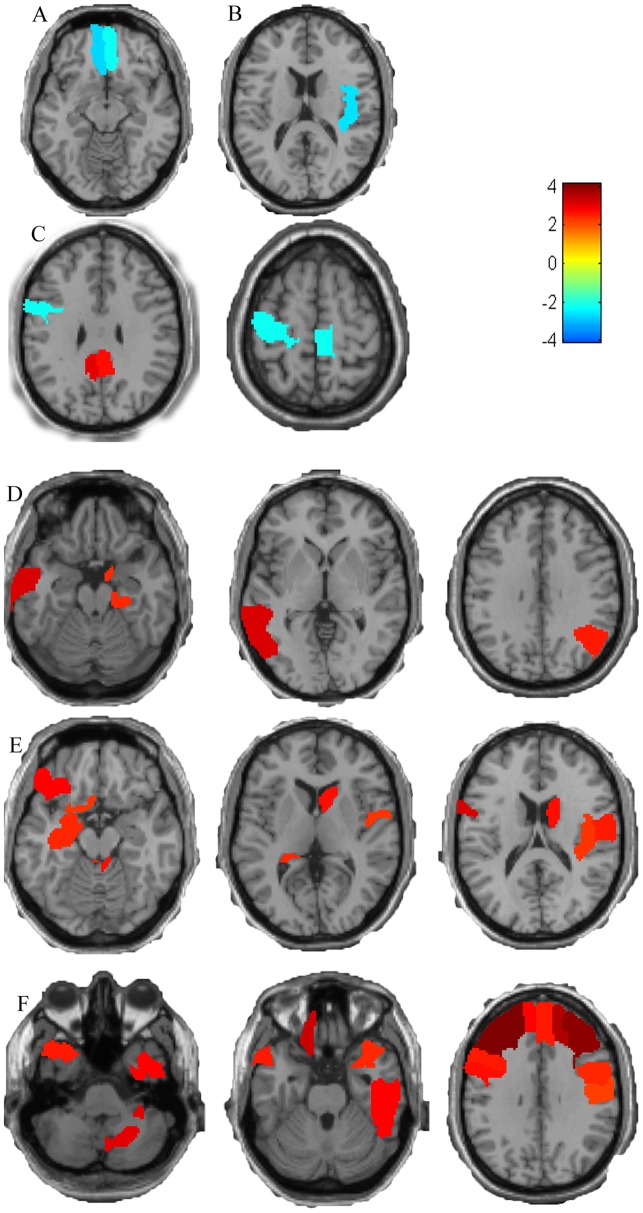
The brain regions which have been selected as features more than 23 times. (A) Selected regions with ReHo. (B) Selected regions with ALFF. (C) Selected regions with GM. (D) Selected regions with RFCS. (E) Selected regions with WM. (F) Selected regions with CSF.

**Table 5:** The brain regions which have been selected as features more than 23 times.

Type	Region	P-value	T-value
ReHo	ORBmid_L	0.0281	−2.2710
	ORBmid_R	0.0092	−2.7258
ALFF	ROL_L	0.0121	−2.6164
RFCS	PHG_L	0.0295	2.2504
	ANG_L	0.0244	2.3316
	MTG_R	0.0044	3.0034
GM	PreCG_L	0.0297	2.2469
	PCG_L	0.0166	2.4896
	PCG_R	0.0071	2.8237
	PCL_L	0.0273	−2.2825
WM	PreCG_R	0.0044	3.0050
	ORBinf_R	0.0137	2.5674
	ROL_L	0.0383	2.1359
	OLF_R	0.0415	2.1003
	HIP_R	0.0332	2.1990
	AMYG_R	0.0290	2.2580
	PoCG_L	0.0216	2.3826
	CAU_L	0.0174	2.4709
	CER3_L	0.0163	2.4989
	Ver1_2	0.0280	2.2719

A positive t-value represents increased values in the PD group. Abbreviations: R, right; L left.

Structural images were preprocessed using SPM8. We first implemented bias correction for all of the images [14]. Segmentation and normalization were performed using the default tissue probability maps. All the images were then re-sampled to an isotropic resolution of 3 mm at the end of the normalization and segmentation process to maintain a constant resolution across all of the subjects. All images were modulated so that the total amount of tissue density in the modulated image remained similar to that of the original image. The modulated images then underwent spatial smoothing by a 10-mm full width at half maximum (FWHM) Gaussian kernel.

### Feature extraction

ALFF is an effective indicator of intrinsic or spontaneous neural activity in the human brain [16]. We calculated the ALFF as follows. The time series of each voxel was first converted into the frequency domain using a Fast Fourier Transform, and the power spectrum was then obtained. The square root was calculated at each frequency of the power spectrum, and the ALFF was calculated as the mean of this square root [16]. To reduce the global effects of variability in all subjects, the ALFF of each voxel was divided by the global mean value. Thus, an ALFF map was obtained for each subject. The ALFF map was then partitioned into 116 regions of interest (ROIs) according to the Automated Anatomical Labeling (AAL) atlas (Table 2) [17], and the mean ALFF of each region was calculated by averaging the ALFF values within that region. The ALFF feature of one subject consisted of the mean ALFF of every region.

The ReHo measures the functional synchronization of a given voxel with its nearest neighbors and can be used to evaluate brain activities in the resting-state [18]. The ReHo of one time series is defined as the Kendall's coefficient of the time series of a given voxel and those of its nearest neighbors [19]. The number of neighboring voxels is 26. To reduce the global effects of variability in all of the subjects, the ReHo of each voxel was divided by the global mean ReHo value for each subject. Thus, a ReHo map was obtained for each subject. The individual ReHo map was then partitioned into 116 ROIs using the AAL template, and the mean ReHo of each region was calculated by the average of the ReHo values within that region. The ReHo feature of one subject consisted of the mean ReHo of every region.

The RFCS measures the average correlation strength of a given region compared with all of the other regions. We first regressed out the effects of head motion and the whole brain averaged signal [20]. The individual volume was first partitioned into 116 ROIs using the AAL atlas, and the mean time series of each region was then extracted by averaging the time series within that region. Each subject was assigned a 116×116 correlation matrix. This matrix was calculated using the Pearson correlation coefficients between the time series of all the potential pairs of regions. We then measured the RFCS using a method described in our previous study [21]. The RFCS of region i was defined as:
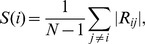
where R_ij_ is the correlation coefficient between region i and region j and N is the number of regions.

As a result of preprocessing, the volume of each subject was divided into three images: the GM image, the WM image and the CSF image. Similar to the functional maps, the individual modulated images were first partitioned into 116 ROIs using the AAL atlas, and the mean value of each region was then extracted by averaging the values of all of the voxels within that region. Thus, we extracted 116 features separately from the GM, WM and CSF maps for each subject.

### Feature selection

Because the extracted features included considerable decreasing classification accuracy and the generalization of “noise”, we used the feature selection algorithm to select features with the most discriminative ability. In this study, the feature selection method was used to compare the feature values of the various brain regions between the two subject groups. Features with significant differences (P<0.05, uncorrected) between the two groups were selected. Two-sample t-tests were then performed to determine the features that showed differences between the PD and normal control (NC) groups. We also used a nonparametric rank-sum test for the feature selection and obtained similar results. The remaining feature values for each subject were then concatenated into a single vector.

### Classification algorithm for PD and NC

In this study, we used supervised learning methods to construct the classifier. Briefly, a supervised machine-learning algorithm was “trained” to produce a desired output from a set of input (training) data. The supervised machine learning algorithm used in this study is was the support vector machine (SVM) [22]. The algorithm was developed using MATLAB (The Math Works, Natwick, MA) and LIBSVM (http://www.csie.ntu.edu.tw/~cjlin/libsvm/). The leave-one-out cross-validation (LOOCV) method was used to estimate the performance of the classifier. To train the SVM classifier, the selection of a penalty parameter C and kernel function parameters G were very important. The grid search was performed over the range C = [2^−10^, 2^−9^... 2^9^, 2^10^] and G  = [2^−10^, 2^−9^,...., 2^9^, 2^10^]. The optimized C and G were then used to create the optimized SVM model. A hyperbolic tangent function was selected as the kernel function in this study. The output of this model was an abnormality index-score.

## Results

We used the LOOCV to estimate the generalization ability of the classifier. Our multi-model method achieves a classification accuracy of 86.96%, with a sensitivity of 78.95% and a specificity of 92.59%. These results were better than the values obtained using the single-model feature combinations. The classification performance of the combined features is listed in Table 3. We also list the number of features retained using this method per fold in Table 4.

Using each subject's abnormality index-score as a threshold, the performance of the receiver operating characteristics (ROC) curve is shown in Fig. 2. The area under the ROC curve (AUC) of the proposed method is 0.951, which indicates excellent diagnostic power. Figure 3 is scatter plot of the abnormality index-scores of all subjects.

Table 5 lists the brain regions which have been selected as features more than 23 times (Fig. 3). Compared to the NCs, the PD patients showed significant ReHo value decreases in the bilateral ORBmid, ALFF decreases in the left ROL and significant RFCS increases in the left PHG, left ANG and right MTG. Moreover, the volume of the GM in the left PCL showed significant decreases, and the left PreCG and the bilateral PCG showed a significant increase in GM volume in the PD group. Brain regions showing WM volume changes were mainly located in the frontal and temporal lobes. Additional details are described in Table 5 and Figure 4.

## Discussion

A clinical pathological study showed that the diagnostic accuracy for idiopathic PD (IPD) was approximately 75% and that the remaining 25% accounted for those diagnosed with IPD who exhibited progressive supranuclear palsy (PSP), multiple system atrophy (MSA), Alzheimer's disease, or basal ganglia vascular disease post mortem [23]. Analysis based on neuroimaging (PET or SPECT) can improve this diagnostic accuracy to approximately 90% for PD patients [6], [7], [24]. However, these imaging modalities are invasive and are not suitable as routine diagnostic tools. In the present study, using a combination of both functional and structural imaging technology, we constructed a non-invasive, multi-modal magnetic resonance imaging algorithm framework for the diagnosis of early PD. The study provides evidence that the method can be developed to correctly differentiate between PD and NC.

Compared with a single modality, the advantage of using multiple modalities is to extract more features (effective features). In this study, machine learning methods are used to construct classifier. Theoretically speaking, multiple modalities method adopts different features as inputs; these inputs respectively reflect different aspects of samples. We believe that this strategy of feature selection for issue classification reflects more profiles of different classes and will be able to obtain more accurate solution. In fact, my results show that the performance of classifier based on single modality is lower than the multi-modal method classifier (see Table 3). There were two modalities to be used: one was structural imaging, the other was functional imaging. For the rsfMRI images, we extract features from three different perspectives: ALFF, ReHo and RFCS. ALFF has been proven to be a effective tool for evaluation of PD [25], [26]. ReHo in cerebellum was positively correlated with UPDRS [8]. Effective connectivity between cerebellum and frontal lobe was negative correlated with UPDRS [27]. It shows that they have different perspectives; the information obtained is not the same. Because we want to extract useful information as much as possible, all three indicators are used.

An important purpose of this study is to find effective biological indicators which can represent differences between the early PD and NC. In recent years, in pattern recognition based neuroimaging, structural images were often used to diagnose neuropsychiatry disease, such as Alzheimer's disease [28], [29], [30] , mild cognitive impairment [31], [32], Huntington's disease [33] and schizophrenia [34]. But few studies have reported diagnosis of PD according to the structural images. The main reason may be that brain structure changes in PD patients is still a controversial issue. Some studies showed there was no structural difference between PD and NC [35], [36] and others showed PD patients had atrophy in brain stem [37],frontal gyrus [38], [39], temporal gyrus [40] and insula [38], [40]. There are many reasons for this result. The most likely reason is that the VBM method is voxel-based and it can not detect changes in the structure of the brain regions. Thus, we used a template-based approach to find structural changes of the various functional areas of the PD patients. From the results, some functional areas do occur structural changes which may reflect the physiological changes of PD. We found the volume of precentral gyrus was increased, consisted with one previous study [41]. Some studies have showed an increased activation in precentral gyrus in PD [42], [43]. Further studies demonstrated that the functional connectivity of precentral gyrus was increased in motor network in PD [44], [45]. The strengthened functional connectivity may also reflect a facet of the primary pathophysiology of PD, due to an inability to inhibit contextually inappropriate circuits [45], [46].

Through feature selection, we selected brain regions with the most discriminative power between PD and NC subjects. These results provided some valuable clues for the early diagnosis of PD. For example, we found that the ReHo of the bilateral ORBmid had declined, which is consistent with the findings of previous studies [8]. The prefrontal cortex is a part of the associative circuit in the striatal-thalamo-cortical loops [47]. One previous study has shown that cognitive decline in PD is related to a reduced 18F-fluorodopa uptake in the associative circuit [48]. Thus, the assessment of both the motor and non-motor symptoms may be important for the early diagnosis of PD.

Our research used a template-based approach to extract the features, and we did not use the traditional region of interest (ROI) method for three main reasons. First, several studies have shown that functional changes in PD patients–not only activity changes in several brain areas [8] but also higher levels of change, such as functional connectivity changes [44] or brain network changes [21]–may be effective biological markers to identify PD pathological changes. Thus, the ROI method is not appropriate for the proper qualification of these changes. Second, based on previous fMRI studies [8], [43], [49], [50], we found conflicting results regarding changes in the activities of some brain regions. Thus, it is difficult to define brain regions that truly represent pathological changes in PD. Third, because we want to build the automatic diagnosis system, identification of ROI required human intervention and therefore not suitable for use in this method.

Two brain templates were selected: the 116-brain regions template and the 90-brain regions template. The difference between the two templates was that the former contained 26 cerebellar regions. One previous study showed that the ReHo of the cerebellum in PD patients increased in the resting-state [8]. Several other studies showed that the functional connectivity [44] and effective connectivity [27] of the cerebellum increased when the patient performed a movement task. In addition, it has been proposed that hyperactivation of the cerebellum in PD patients is a functional compensation for a defective basal ganglia [51], [52], [53]. Thus, we used the template of 116 brain regions to extract features in this study.

There are several areas in need of improvement in this study. First, we used a linear regression method to reduce the effects of the low-frequency drifts and the high-frequency physiological noise; however, this was not the most effective approach. In future studies, these physiological effects should be estimated and removed by simultaneously recording the respiratory and cardiac cycles during the data acquisition process. Second, although we used both structural MRI and resting fMRI data, there are also other modalities (e.g., diffusion tensor imaging) that may be used to further improve the classification performance. DTI has been suggested as a potential method for diagnosis of PD [54]. We also use MNI template to extract FA features and combine it with other features. It doesn't improve the final classification results. The probable reason is that,due to the impact of registration error, extraction of effective DTI features by my method becomes a very difficult thing. Further work is to develop new technique to extract DTI features. Third; we used the AAL atlas to divide the brain into 116 ROIs. Other structural [55] and functional [56], [57] brain atlases may also be used, as different segmentation methods may generate different results. In fact, several recent studies have demonstrated that the connectivity patterns of brain networks can be affected by different parcellation atlases [57], [58]. Future studies should apply our method to other brain atlases. Fourth, because a small sample (46 subjects in total) was used in this study, the obtained classifier is specific to the current dataset and may not be applicable to other datasets. In the future, we would like to use a larger dataset to determine the generalizability of this method.

### Conclusions

In this study, we developed a method used to distinguish patients with early PD from NCs using a combination of multi-modal imaging and multi-level measurements. The discriminative power of this method was very high, yielding an accuracy of 86.96%. This promising classification power suggests that this method may provide a non-invasive approach that may improve the clinical diagnosis of early PD.
